# Usefulness of a novel density measurement drill for evaluating cancellous bone density: correlation between CT value and drilling torque value in bovine ribs

**DOI:** 10.1186/s40729-025-00596-9

**Published:** 2025-01-31

**Authors:** Kazuya Doi, Kaien Wakamatsu, Reiko Kobatake, Yoshifumi Oki, Yusuke Makihara, Masaru Konishi, Kazuhiro Tsuga

**Affiliations:** 1 Department of Advanced Prosthodontics, Hiroshima University Graduate School of Biomedical and Health Sciences, 1-2-3 Kasumi, Minami-ku, Hiroshima City, 734-8553 Hiroshima Japan; 2 Department of Oral and Maxillofacial Radiology, Hiroshima University Hospital, 1-2-3 Kasumi, Minami-ku, Hiroshima City, 734-8551 Hiroshima Japan

**Keywords:** Bone density, Dental implant, Multidetector computed tomography, Misch classification, Intraoperative evaluation

## Abstract

**Purpose:**

The study aimed to examine the usefulness of a novel density measurement drill for evaluating cancellous bone density by examining the correlation between computed tomography (CT)-based Misch bone density classification and drilling torque value.

**Methods:**

Bovine ribs were used as the drilling sites for implant placement. Multidetector CT (MDCT) was performed after contrast materials were attached to the drilling sites. CT value within the region of interest (ROI) on MDCT scan was measured and classified according to the Misch classification (D1 to D5). Drilling torque value was measured using a novel measurement drill. Next, histomorphometric analysis of the drilling site was performed to assess bone density, expressed as percentage of bone area within ROI.

**Results:**

MDCT showed the presence of D2 (*n* = 87), D3 (*n* = 92), D4 (*n* = 133), and D5 (*n* = 52) at the measurement sites, however, no sites were classified as D1. The drilling torque values were 11.2 ± 3.2 Ncm for D2, 7.8 ± 3.3 Ncm for D3, and 3.0 ± 1.2 Ncm for D4, and 1.4 ± 0.6 Ncm for D5, with significant differences. A positive correlation was observed between CT value and drilling torque value (*r* = 0.99). Histomorphometric analysis revealed a positive correlation between drilling torque value and bone area ratio (*r* = 0.97).

**Conclusions:**

The results of this limited study demonstrated the usefulness of the direct and objective cancellous bone density evaluation method using a novel measurement drill. This evaluation method will be informative for subsequent treatment decisions.

**Supplementary Information:**

The online version contains supplementary material available at 10.1186/s40729-025-00596-9.

## Introduction

Primary implant stability is key to achieving osseointegration and long-term implant success [1, 2]. Bone density at implant placement sites is an important factor affecting primary implant stability [3], and it is currently assessed preoperatively using radiographic examinations such as panoramic radiography and computed tomography (CT), which is an indirect evaluation. There have been reports on bone density classification for implant treatment [4–6]. The Lekholm and Zarb classification categorizes bone into four groups based on subjective evaluation of cortical bone width and trabecular bone density [4]. The Misch classification categorizes bone into five groups, D1–D5, based on the combination of the surgeon’s tactile perception during implant site drilling and the radiographic assessment of the CT value expressed in Hounsfield units (HU): D1 (> 1250 HU), D2 (850–1250 HU), D3 (350–850 HU), D4 (150–350 HU) and D5 (< 150 HU) [5, 6]. These values represent the average X-ray attenuation at each pixel in the CT image. The scale is defined as 0 HU for water and − 1000 HU for air. The CT value can be evaluated by multidetector CT (MDCT); however, these instruments are only available at limited facilities and impose a higher radiation exposure, limiting the widespread use of MDCT for assessing bone density [7–9]. Intraoperative evaluation allows for the direct evaluation of the bone at the implant site; however, this is a subjective evaluation based on the tactile properties judged by the operator. A review article presented the method for evaluating bone density by measuring the resistance torque when drilling bone with a specific probe as intraoperative measurement of bone density [10]. Torque measurements during drilling have been proposed as an objective intraoperative evaluation [11–15]. These studies used φ3.0-mm drills for measurement, which potentially limit the subsequent treatment decisions such as drilling protocol and implant size. Thus, making treatment decisions based on intraoperative evaluations without such a limitation requires a measurement drill with a narrow diameter. We developed a novel density measurement drill, which is effective for torque detection despite its narrow diameter [16]. The relationship between CT-based Misch bone density classification and torque value during drilling with such a kind of measurement drills have not been clarified. We hypothesized that the torque value obtained using the novel density measurement drill was correlated with the Misch bone density classification.

The study aimed to examine the usefulness of a novel density measurement drill for evaluating cancellous bone density by examining the correlation between CT-based Misch bone density classification and drilling torque value.

## Methods

### Samples and measurement devices

This study used bovine ribs with cortical and cancellous bone regions. The bovine ribs were obtained from the Hiroshima City Central Wholesale Market as bone blocks after processing, and then stored frozen at -20℃. Before the experiment, the bone blocks were thawed and immersed in 0.9% saline.

**Fig. 1:**
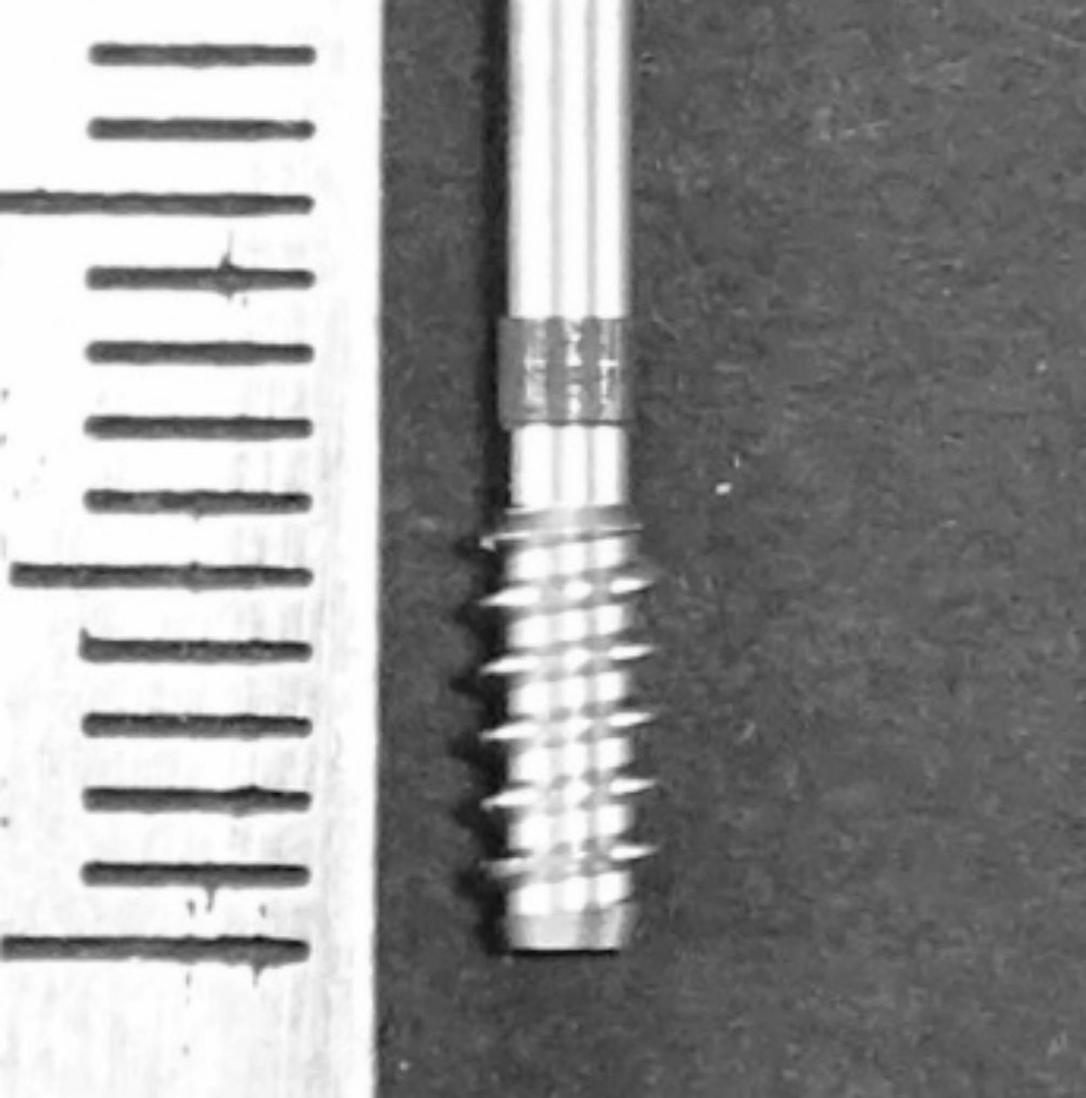
Novel density measurement drill. The drill has a spiral structure blade and a cylindrical shape with a flat tip (Outer diameter 2.7 mm). The drilling length (below the black line) is 7.0 mm

Bovine ribs have cortical and cancellous structures similar to those of the human maxilla and mandibular bone, and they have previously been used to evaluate bone structure [11]. The drilling torque value was measured using a novel density measurement drill (Nakanishi Inc., Tochigi, Japan) (Fig. 1), which we have previously reported to be useful for torque measurement despite its narrow diameter [16]. The drill diameter is 2.7 mm, which is the maximum diameter including the threads. This drill was designed with a spiral structure blade and cylindrical shape with a flat tip to be useful for torque detection. The drilling torque values were recorded using a computerized surgical implant motor device (Surgic Pro2; Nakanishi Inc.), which can detect torque values of ≤ 1.0 Ncm.

### CT evaluation

**Fig. 2:**
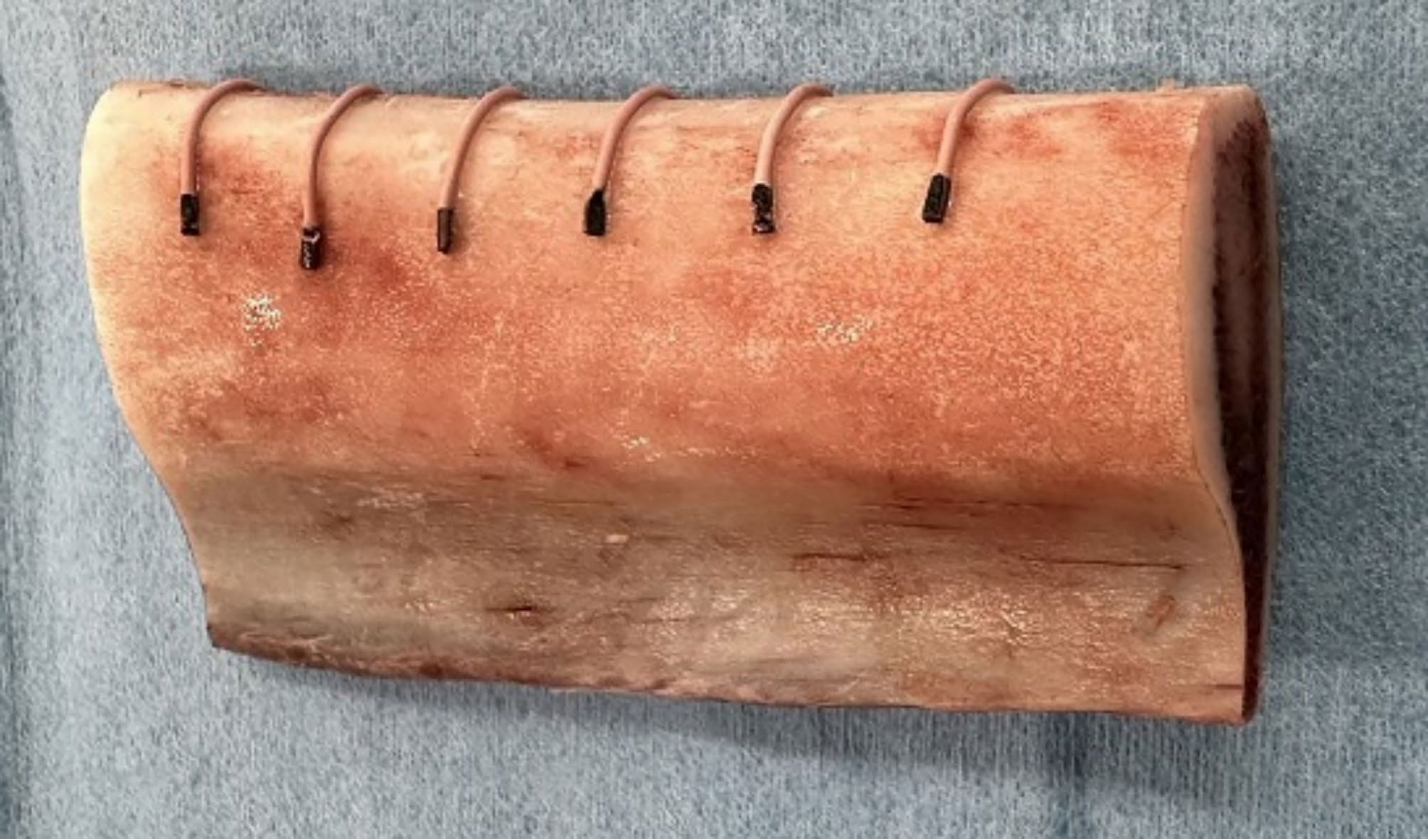
Bovine rib marked with contrast materials. The measurement sites are set every 1–2 cm

**Fig. 3:**
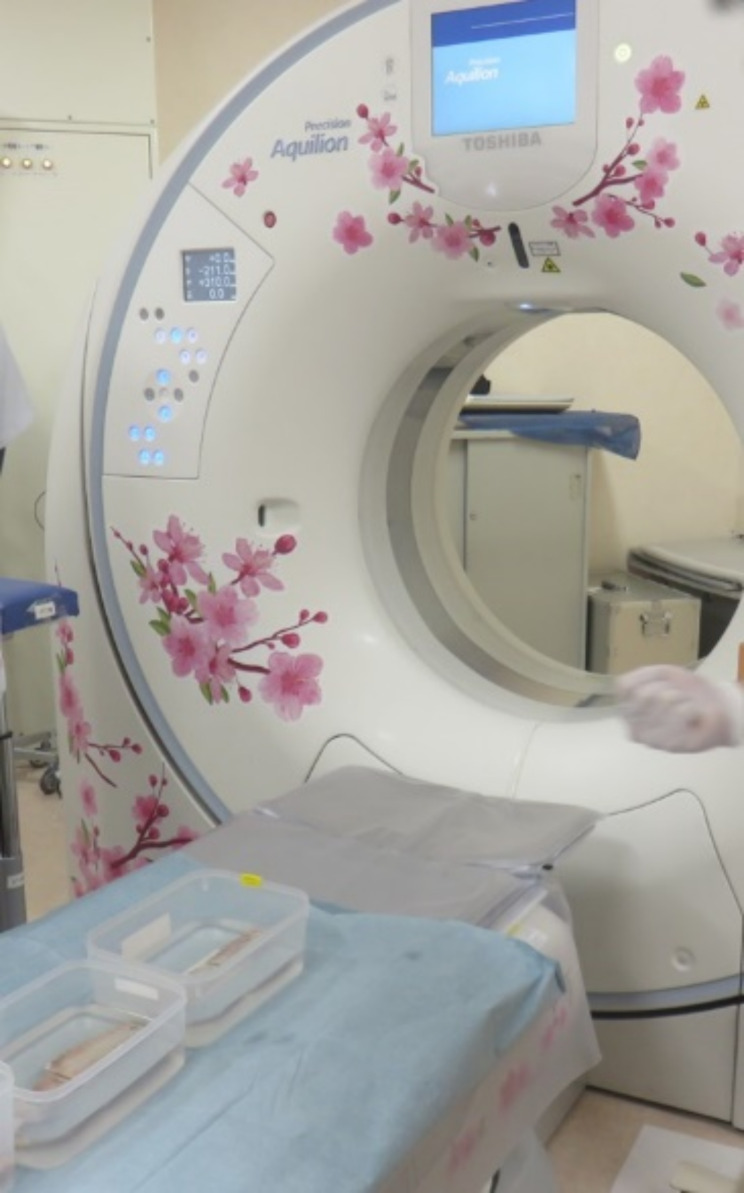
Multidetector computed tomography. Scan was performed with the bovine ribs immersed in 0.9% saline

**Fig. 4:**
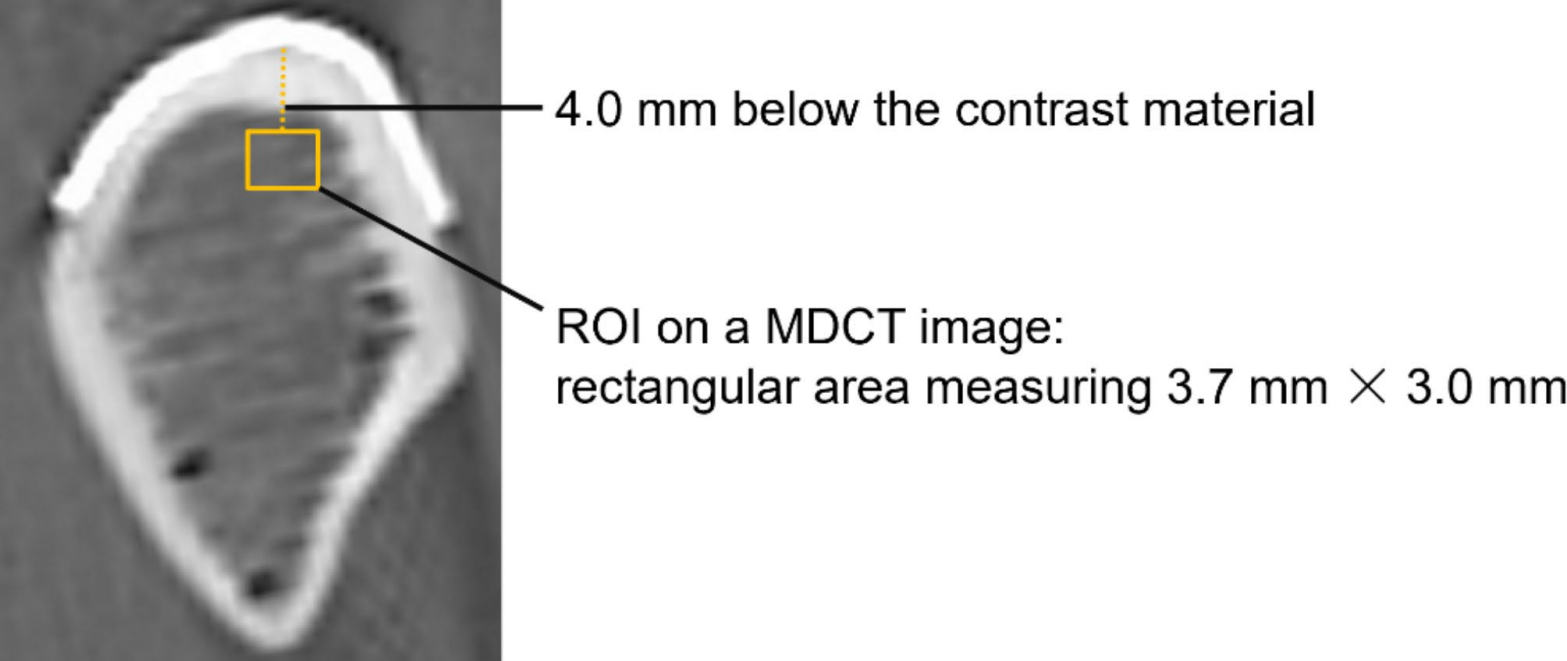
Region of interest (ROI) on a multidetector computed tomography (MDCT) image ROI is defined as the rectangular area (yellow line) located 4.0 mm below the contrast material

The bovine ribs were divided into several segments, and the periosteum was removed. On each rib segment, contrast materials (gutta-percha points) were attached every 1–2 cm to mark the drilling sites (Fig. 2). The ribs were then immersed in 0.9% saline, and MDCT (Aquilion Precision; Canon Medical Systems Corp., Tochigi, Japan) scan was performed with tube voltage of 120 kV, tube current of 80 mA, and a collimation of 80 × 0.5 mm (Fig. 3). Scan data were then imported into the reconstruction software (SYNAPSE VINCENT; FUJIFILM Corp., Tokyo, Japan) and the CT value was measured on the cross-sectional image at each marked site. The region of interest (ROI) on a MDCT image was defined as a rectangular area measuring 3.7 mm × 3.0 mm (Fig. 4). The horizontal dimension of the ROI was in the range of 3.7 mm centered on the implant planning site, including 0.5 mm on both sides of the novel density measurement drill. The vertical dimension was in the range of 4.0–7.0 mm from the contrast material (length: 3.0 mm). As the drilling torque value is significantly affected by lateral force, the lower area of the implant bottom was excluded from the measurement site. The average CT value within the ROI was used for evaluation. Each marked site was categorized into the Misch classification of D1–D5 based on CT value.

### Drilling torque measurement

**Fig. 5:**
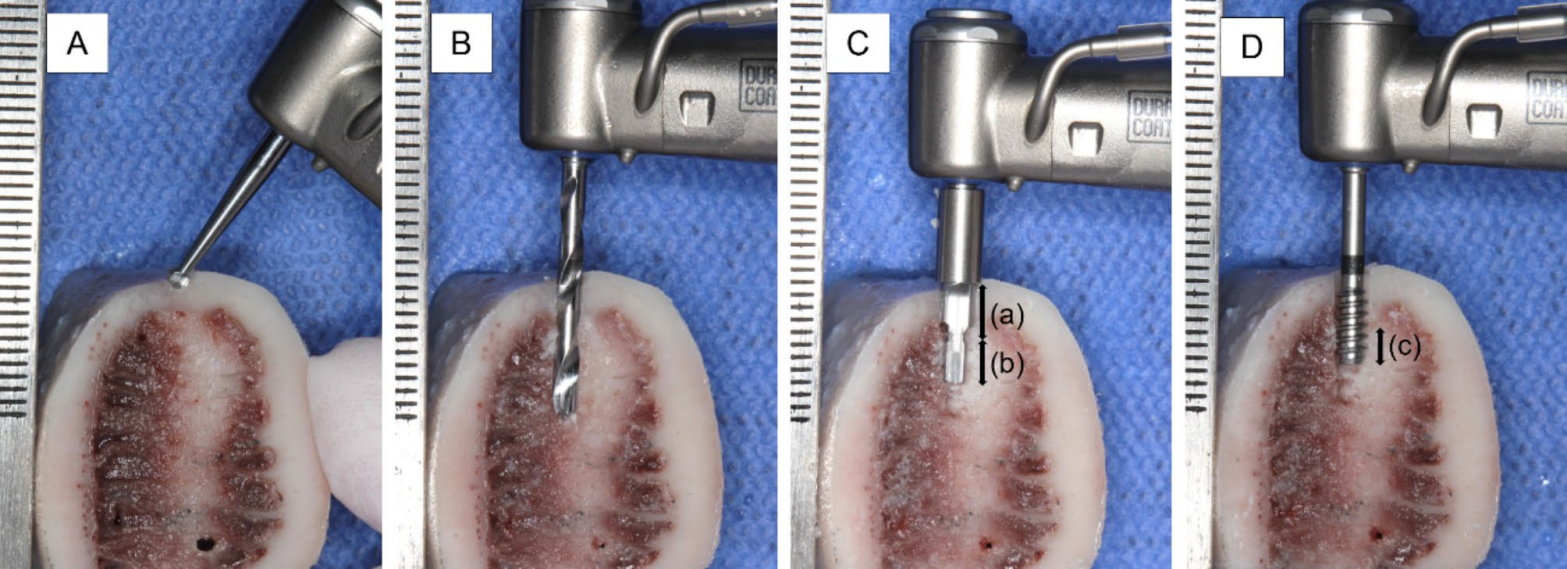
Drilling procedures and measurement of drilling torque value Shown are central cross-sections of the drilling site. (**A**) Round drill (φ2.0 mm) was used to determine the drilling site. (**B**) Twist drill (φ2.0 mm) was used to drill up to 10 mm. (**C**) Pilot drill (a: upper part φ2.8 mm length 4.0 mm, b: bar tip part φ2.0 mm) was used to drill up to 4.0 mm to completely remove cortical bone area. Bar tip part did not affect the measurement socket prepared in the previous step. (**D**) Novel density measurement drill (φ2.7 mm) was used to measure the drilling torque value(c: measurement part φ2.7 mm length 3.0 mm).The drilling torque value is measured at the cancellous bone area

The drilling torque value was measured at each marked site as follows. The drilling procedure is shown in Fig. 5. First, a φ2.0-mm round drill was used to determine the drilling site. Drilling was then performed with a φ2.0-mm twist drill up to 10 mm to avoid picking up torque hitting the bottom of measurement drill. Then, a φ2.8-mm pilot drill was used to remove the upper 4.0 mm of the bone to completely remove the cortical bone area. The bar tip part of pilot drill is 2.0 mm in diameter, the same diameter as twist drill, and determined the drilling direction. The drilling torque values were measured using a φ2.7-mm novel density measurement drill up to 7.0 mm (measurement depth: 3.0 mm), and the peak torque value was recorded. The 3.0-mm measurement depth corresponded to the vertical length of the ROI on the CT image. The drilling preparation steps were performed at 1200 rpm, whereas the drilling torque was measured at 35 rpm. All steps were performed under continuous 0.9% saline irrigation. Each measurement was performed by five experienced operators randomly (*n* = 364).

### Histological evaluation

After measuring the drilling torque value, four sites with approximately the median CT value for each Misch classification, respectively, were selected.

The observation section was set at the central cross-section of the drilling site.

The selected tissue blocks were fixed in 10% neutral formalin, and decalcified with hydrochloride solution (KC-X^®^; FALMA, Tokyo, Japan). Then, dehydrated using a graded ethanol series, cleared with xylene, and embedded in paraffin. Sections with 5 μm thickness were obtained from each block and stained with hematoxylin and eosin. Histological analysis was performed using light microscope (BZ-9000; Keyence, Osaka, Japan). Histological images were digitized and histomorphometrically analyzed using ImageJ (National Institute of Health, Maryland, USA) to determine bone density, expressed as percentage of bone area within ROI.

### Statistical analysis

Drilling torque values were assessed using Kruskal–Wallis test followed by Dunn’s post-hoc test. Spearman’s rank correlation coefficient was used to investigate the relationship between CT and drilling torque value, as well as between drilling torque value and bone area ratio. The calculations were performed using Prism v7 (GraphPad, La Jolla, CA). All data are expressed as mean ± standard deviation. In all cases, statistical significance was defined as a *p*-value of < 0.05.

## Results

### CT and drilling torque values

**Fig. 6:**
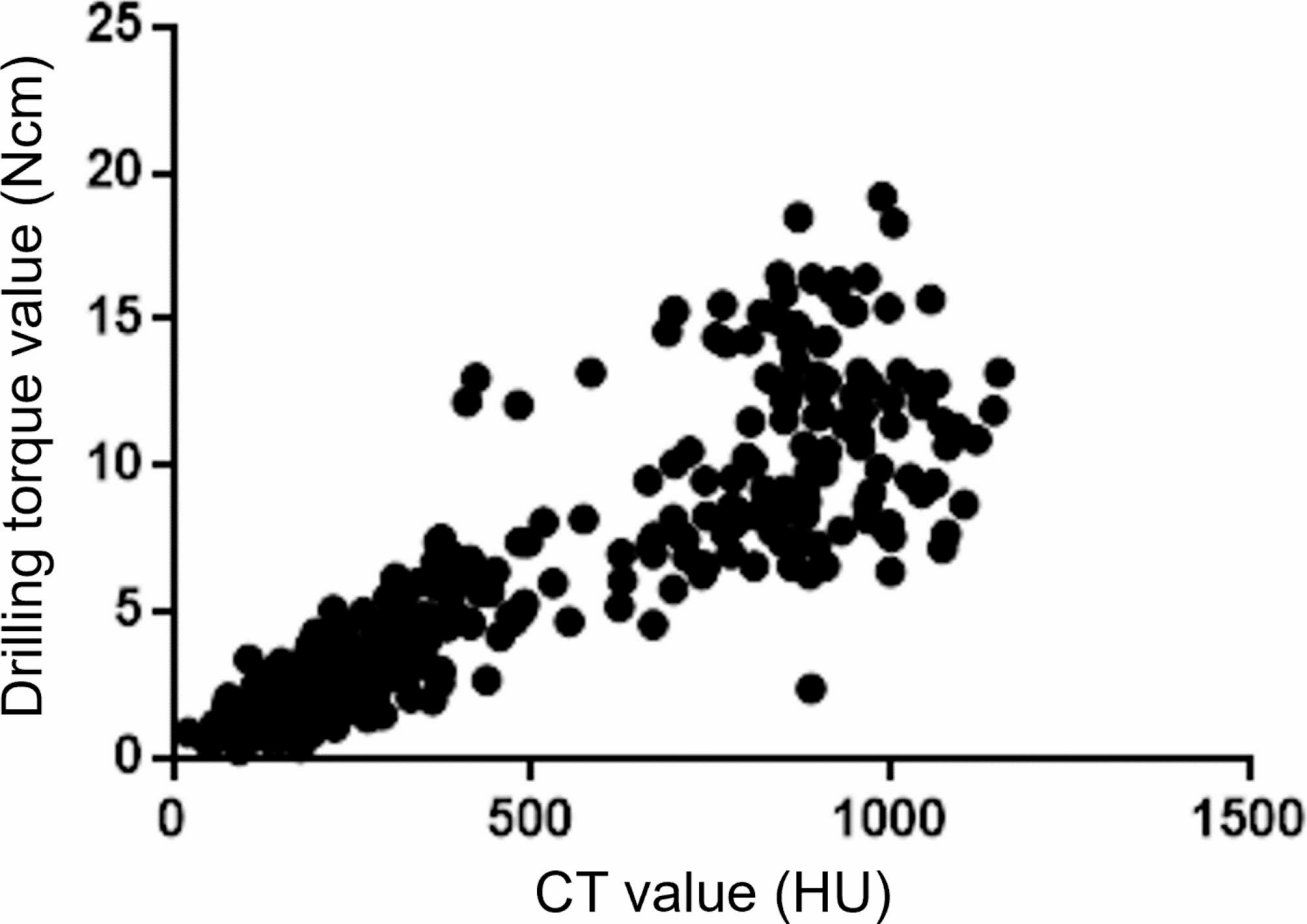
Computed tomography (CT) and drilling torque values Scatterplots demonstrate the significant positive correlation between CT and drilling torque values (*r* = 0.99)

The ROIs on CT scans showed no cortical bone areas or air-containing regions. According to the Misch CT value-based classification, no sites classified as D1 were observed, whereas 87, 92, 133, and 52 measurement sites were classified as D2, D3, D4, and D5, respectively. The drilling torque values of the D2–D5 measurement sites were 11.2 ± 3.2 Ncm, 7.8 ± 3.3 Ncm, 3.0 ± 1.2 Ncm, and 1.4 ± 0.6 Ncm, respectively (Table 1). Their values differed significantly among all classifications and increased with CT value. CT and drilling torque values showed a significant positive correlation (*r* = 0.99) (Fig. 6).

### Drilling torque value and histomorphometric evaluation

**Fig. 7:**
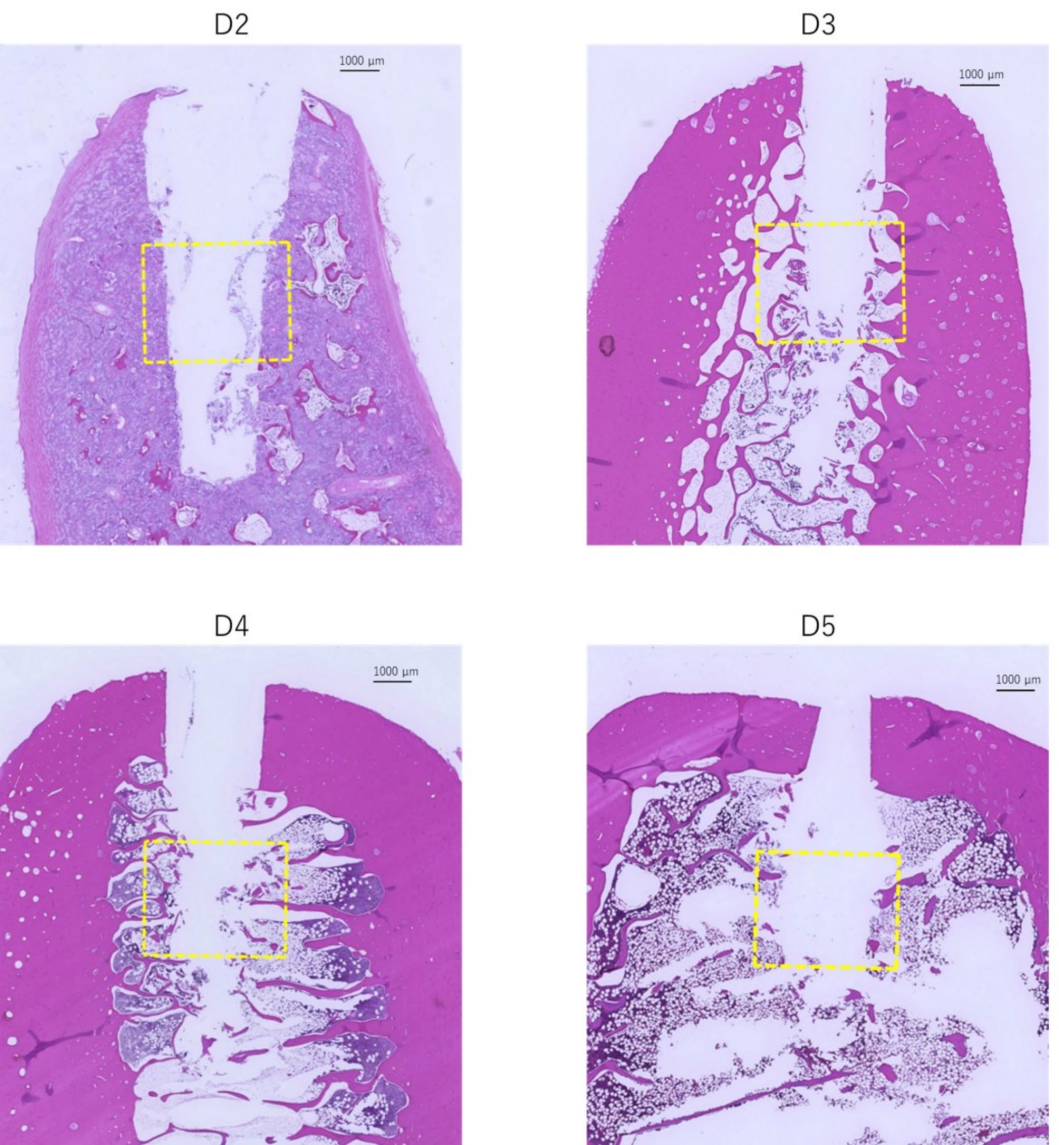
Histological observation of density classification **D2**–**D5**. Histological images including the area around the region of interest (ROI), shown within the yellow dotted line and measuring 3.7 mm × 3.0 mm, after the drilling torque value measurement. (**D2**) Homogeneous and dense osteoid-like structures occupy the area. (**D3**) Dense trabecular bone within the ROI, with the entire bone surrounded by thick cortical bone. (**D4**) A thin trabecular structure and abundant bone marrow area within the ROI. (**D5**) Areas mainly occupied by bone marrow, including a small amount of trabecular bone, within the ROI

**Fig. 8:**
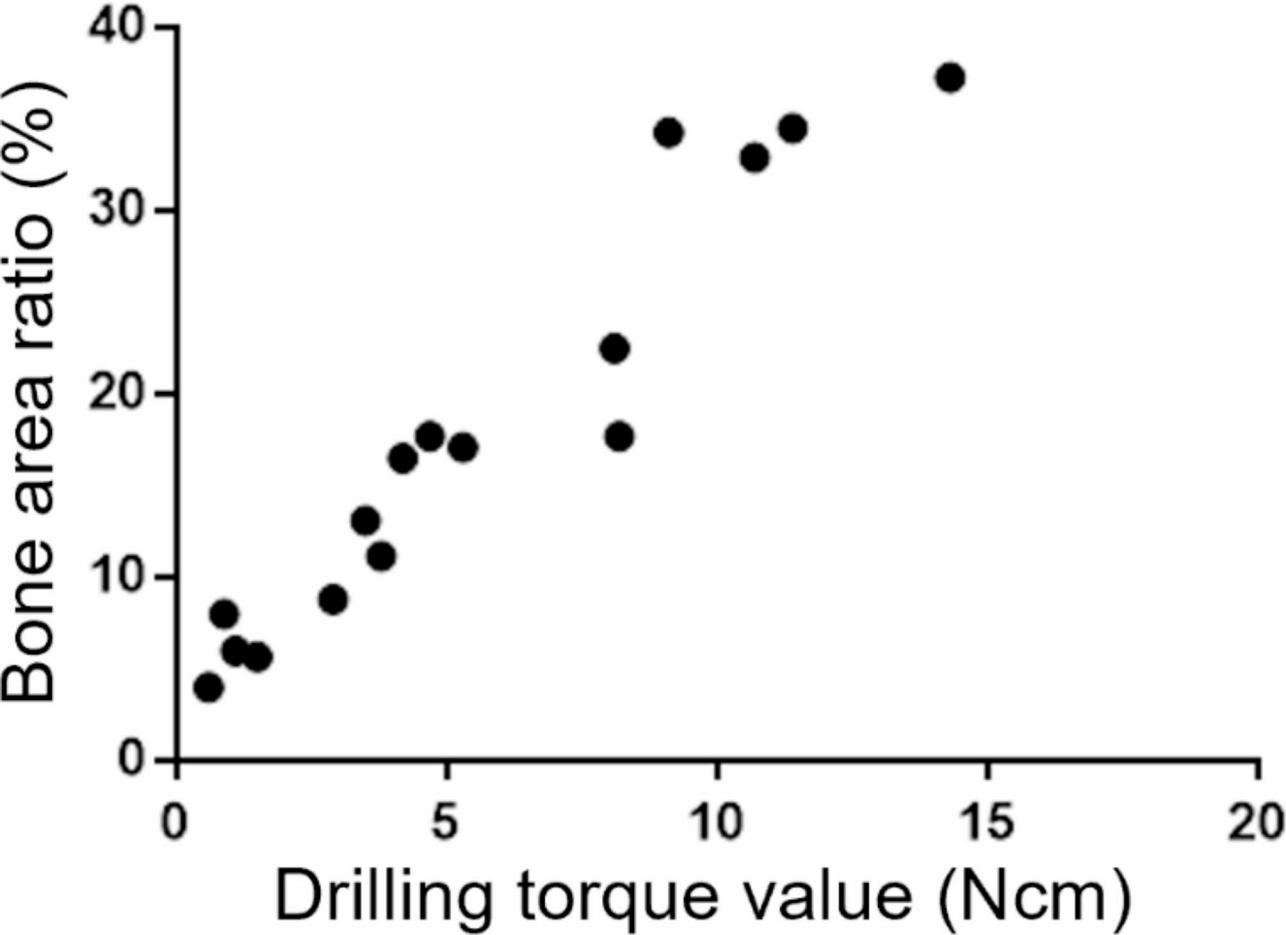
Drilling torque value and bone area ratio. Scatterplots show a significant positive correlation between drilling torque value and bone area ratio (*r* = 0.97)

Representative histological observations of D2–D5 after measuring the drilling torque, including the area around ROI, are shown in Fig. 7. The D2 image showed a homogeneous and dense bone structure, whereas D3 included dense trabecular bone, and the proportion of bone marrow area increased toward D5. The distribution of the drilling torque value and histomorphometric parameter for each sample is summarized in Table 2. The drilling torque value increased with the bone area ratio and was significantly positively correlated with this ratio (*r* = 0.97) (Fig. 8).

**Table 1:** Drilling torque value

Bone density classification	Drilling torque value (Ncm)	*n*
D2 (850–1250 HU)	11.2 ± 3.2	87
D3 (350–850 HU)	7.8 ± 3.3	92
D4 (150–350 HU)	3.0 ± 1.2	133
D5 (< 150 HU)	1.4 ± 0.6	52

**Table 2:** Distribution of drilling torque value and histomorphometric parameter for each bone density classification

Sample No.	CT value (HU)	Bone density classification	Drilling torque value (Ncm)	Bone area ratio (%)
1	1005.5	D2	11.4	34.5
2	973.7	D2	9.1	34.3
3	959.0	D2	10.7	32.9
4	910.4	D2	14.3	37.3
5	572.9	D3	8.2	17.7
6	490.6	D3	5.3	17.1
7	552.8	D3	4.7	17.7
8	516.7	D3	8.1	22.5
9	274.1	D4	3.8	11.2
10	325.9	D4	3.5	13.1
11	306.5	D4	2.9	8.8
12	285.9	D4	4.2	16.5
13	110.4	D5	0.9	8.0
14	48.6	D5	0.6	4.0
15	138.6	D5	1.5	5.7
16	90.9	D5	1.1	6.0

## Discussion

In implant treatment, bone density is involved in the acquisition of primary implant stability and osseointegration after implant insertion, so preoperative and intraoperative bone density evaluation is important for treatment planning. Bone structure consists of cortical bone and cancellous bone. The thickness of the cortical bone thickness has a significant effect on the implant insertion torque and resonance frequency analysis for primary implant stability evaluation. This cortical bone thickness can be easily evaluated using preoperative CT images. Also, cancellous bone can be evaluated by the CT value of MDCT or contrast of CBCT images, however direct and objective numerical evaluation has not been possible in clinical.

In this study, bone density at the implant placement site was evaluated based on the drilling torque value using a φ2.7-mm novel density measurement drill. Our previous study demonstrated that verification using simulated artificial bone blocks has confirmed the distinguish of low-density condition [16]. The difference in structure between the novel density measurement drill and other tap drills is that it does not have a longitudinal groove for discharging cutting chips. Therefore, torque measurement is possible even in low-density condition.

The results of this experiment using bovine bone also showed that it was useful for bone density classification. Determining the bone density at the implant placement site is important for obtaining primary implant stability [3]. Drilling protocols such as undersized drilling techniques are selected to achieve primary implant stability in cases with low bone density, and the loading timing is determined based on the level of primary implant stability [17–20]. In the undersized drilling technique, for example, implant manufacturers recommend drilling with a φ2.8-mm drill for placing a regular-sized implant [21]. Other studies on torque values during drilling used drills with diameters of ≥ 3 mm [11–15]. Therefore, cases with low bone density may preclude the protocol using undersized drilling technique. Measuring bone density using the φ2.7-mm diameter drill allowed the consideration of treatment decisions based on intraoperative evaluation, including changes in drilling protocol and implant size. Several studies have used simulated bone blocks corresponding to Misch classifications, which were classified according to their density [12–15, 22–24]. The simulated bone blocks used in these experiments had a homogeneous dense structure, which differs from the actual bone structure composed of cortical and cancellous bones. Therefore, the present study used bovine ribs, which have a structure and size similar to those of the human maxilla and mandibular bone. In the CT evaluation of the bovine ribs in this study, no samples were classified as D1 according to the Misch classification. The Misch D1 classification is mostly composed of cortical bone, excessive torque may occur in hard bone. To avoid excessive torque, a torque limiter should be set. In this study, a pilot drill was used to drill 4.0 mm from the top of the bone before measuring the drilling torque value; thus, the measurement length was 3.0 mm. The cortical bone thickness varies depending on the site, in which the posterior mandible and maxilla are the thickest and thinnest parts, respectively. In most cases, the effect of the cortical bone while measuring drilling torque value can be eliminated by drilling the upper 4 mm of bone [25]. The drilling torque value correlated with the CT value in the present study, enabling the objective evaluation of the cancellous bone at the actual implant site, and this information was useful for subsequently determining the treatment plan. The evaluation of bone structure on preoperative CT scans is an indirect method, whereas the drilling torque value measurement is a direct evaluation at the implant placement site. Bone density assessments based on drilling sensation vary depending on the surgeon’s experience. Some reports have described the difficulty in distinguishing bone density based on the surgeon’s tactile perception during drilling [26, 27]. Trisi et al. examined the relationship between clinical bone density evaluation by drilling sensation and histomorphometric bone density [26]. They concluded that surgeon’s tactile perception allows to distinguish D1 and D4 with a high reliability, however it is difficult to distinguish between D2 and D3. Rokn et al. have reported the difficulty in distinguishing low-density bones such as between D3 and D4 based on drilling sensation [27]. In contrast, the drilling torque values in the present study distinguished the bone densities for D2–D5. The drilling torque value obtained using the novel density measurement drill was positively correlated with CT value, suggesting that drilling torque value can be used to evaluate bone density. Histological observation showed that D3–D5 classification had a trabecular bone structure, whereas D2 had a homogeneous bone structure and different features. The histomorphometric findings showed a correlation between the drilling torque value and bone area ratio, consistent with the findings reported by Iezzi et al. [11]. They have reported a positive correlation between bone density measured using an implant motor and bone density assessed using histomorphometry. These results indicate that drilling torque values are strongly correlated with the trabeculae bone structure around the drilling site. Intraoperative evaluation using a novel density measurement drill showed a positive correlation with CT value based on preoperative evaluation and a positive correlation with bone area ratio around the drilling site based on histomorphometric evaluation, suggesting that measurement of drilling torque value is a useful evaluation that more accurately reflects the bone condition.

In this study, the correlation between drilling torque and CT values ​​was clarified. A limitation of the results of this study is that they were evaluated in bovine bone, and there is concern about differences from human biological bone in clinical practice. In the future, we would like to practice the study in clinical research and establish a novel cancellous bone density classification based on the measurement of drilling torque value. The establishment of this classification will provide useful intraoperative information to the operator regarding not only the adaptation of undersized drilling technique and change in implant size but also recently reported the adaptation of osseodensification drilling technique [28–30]. We consider that the results are expected to contribute to predictable implant treatment.

## Conclusions

The results demonstrated that measuring drilling torque using a novel density measurement drill is a useful direct and objective method for evaluating cancellous bone density. This novel evaluation method can be used to inform subsequent treatment decisions.

## Electronic supplementary material

Below is the link to the electronic supplementary material.

Supplementary Material 1

Supplementary Material 2

## Data Availability

No datasets were generated or analysed during the current study.

## Abbreviations

CT: Computed Tomography
HU: Hounsfield Units
MDCT: Multidetector Computed Tomography
ROI: Region of Interest
