# Supplementary material for: Usefulness of a novel density measurement drill for evaluating cancellous bone density: correlation between CT value and drilling torque value in bovine ribs
Source: Int J Implant Dent. 2025 Jan 31;11:7. doi: 10.1186/s40729-025-00596-9 (PMC11785876; doi:10.1186/s40729-025-00596-9)
Supplement: Supplementary file 1 — Supplementary Material 1 [file 40729_2025_596_MOESM1_ESM.pdf]

✓ BACKGROUND

The study aimed to examine the usefulness of a novel density measurement drill for evaluating cancellous bone density by examining the correlation between CT-based Misch bone density classification and drilling torque value.

✓ METHODS

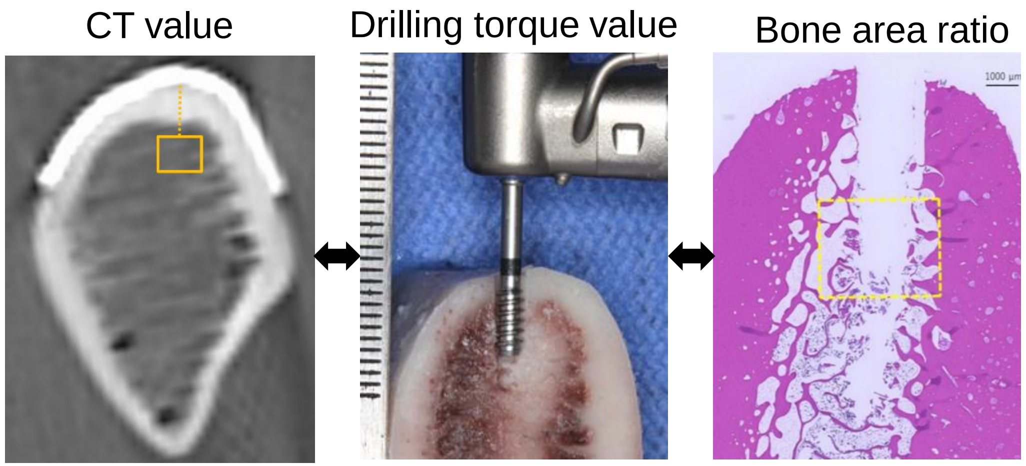

Examining correlation for cancellous bone density

1. Multidetector CT scan of bovine bone
2. Measurement of drilling torque
3. Histomorphometric analysis of the drilling site
4. Examine the correlation

✓ RESULTS

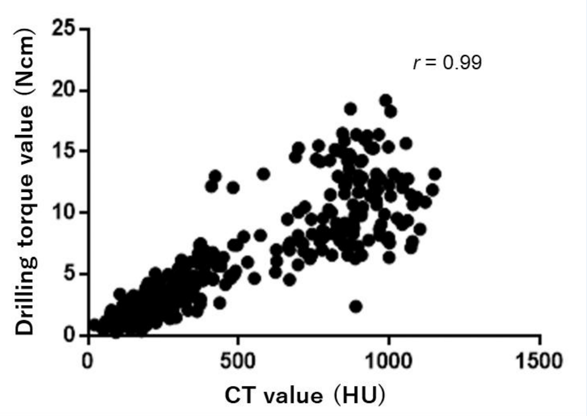

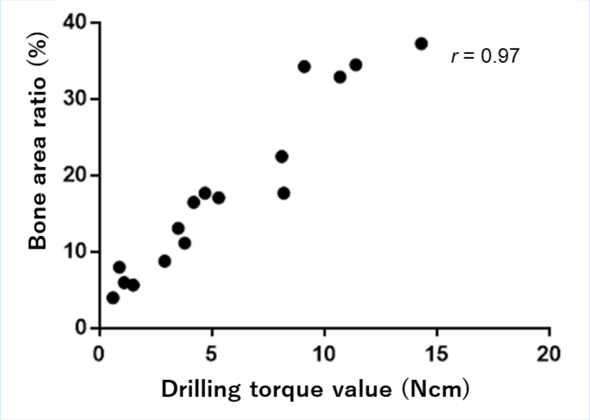

The drilling torque value showed positive correlation with CT value and bone area ratio.

✓ CONCLUSION

The results demonstrated that measuring drilling torque using a novel density measurement drill is a useful direct and objective method for evaluating cancellous bone density.
