# Supplementary material for: Usefulness of a novel density measurement drill for evaluating cancellous bone density: correlation between CT value and drilling torque value in bovine ribs
Source: Int J Implant Dent. 2025 Jan 31;11:7. doi: 10.1186/s40729-025-00596-9 (PMC11785876; doi:10.1186/s40729-025-00596-9)
Supplement: Supplementary file 2 — Supplementary Material 2 [file 40729_2025_596_MOESM2_ESM.pptx]

## Slide 1
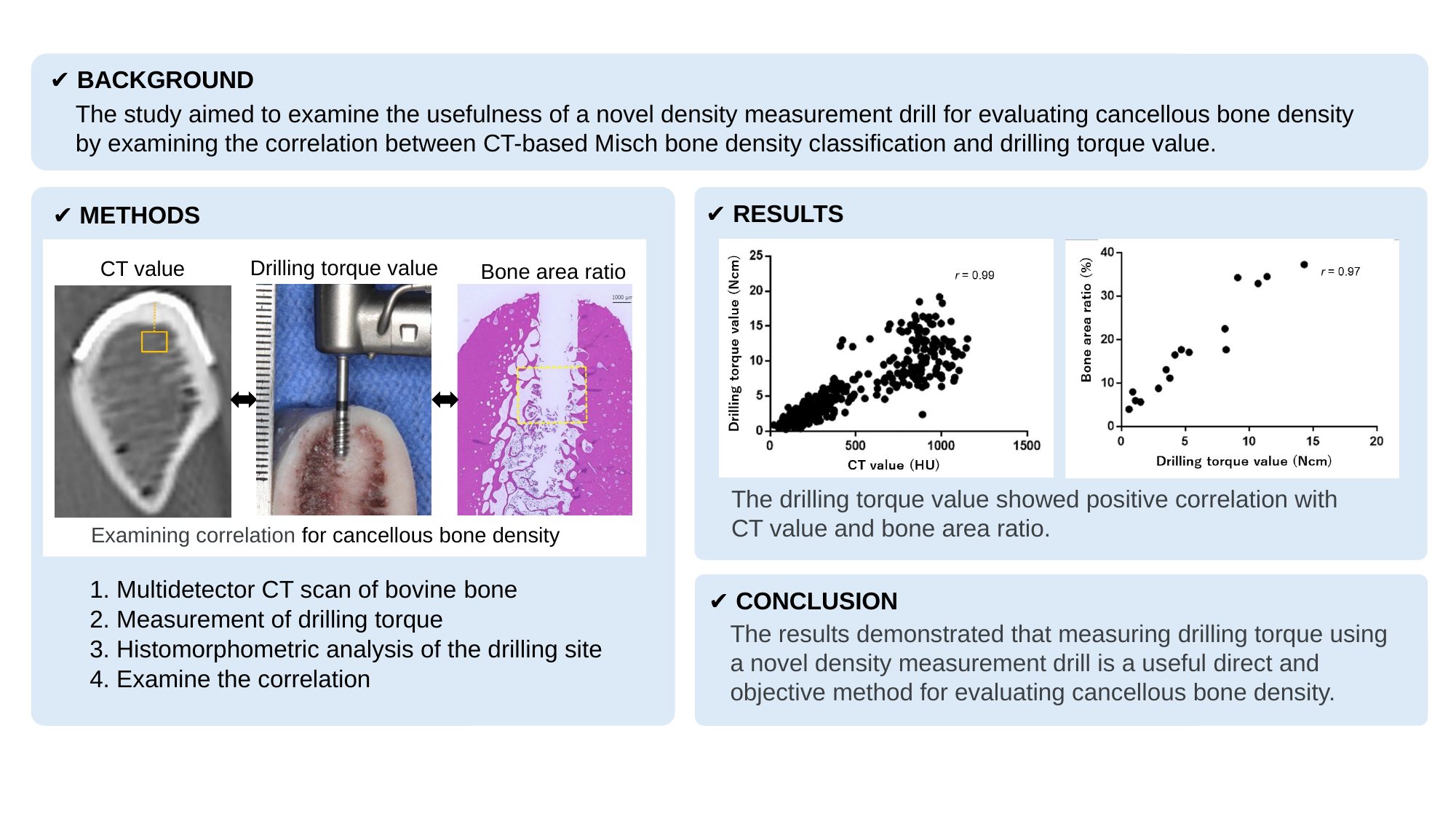

✔ BACKGROUND
The study aimed to examine the usefulness of a novel density measurement drill for evaluating cancellous bone density by examining the correlation between CT-based Misch bone density classification and drilling torque value.
✔ RESULTS
✔ METHODS
Drilling torque value
CT value
Bone area ratio
The drilling torque value showed positive correlation with CT value and bone area ratio.
Examining correlation for cancellous bone density
1. Multidetector CT scan of bovine bone
2. Measurement of drilling torque
3. Histomorphometric analysis of the drilling site
4. Examine the correlation
✔ CONCLUSION
The results demonstrated that measuring drilling torque using a novel density measurement drill is a useful direct and objective method for evaluating cancellous bone density.
